# Granulomatous Prostatitis Mimicking Invasive Prostate Cancer

**DOI:** 10.5334/jbsr.2570

**Published:** 2022-07-06

**Authors:** Romain Gillard, Laurent Médart, Amélie Parisel

**Affiliations:** 1 CHR Liège, BE

**Keywords:** granulomatous prostatitis, prostate cancer, BCG therapy

## Abstract

**Teaching Point:** Benign granulomatous prostatitis mimics prostate cancer on MRI. Peculiar urological history of a patient undergoing prostate MRI helps sorting out that differential, such as a treatment with topic Bacilli Calmette-Guerin instillations.

## Case description

**Figure 1:**
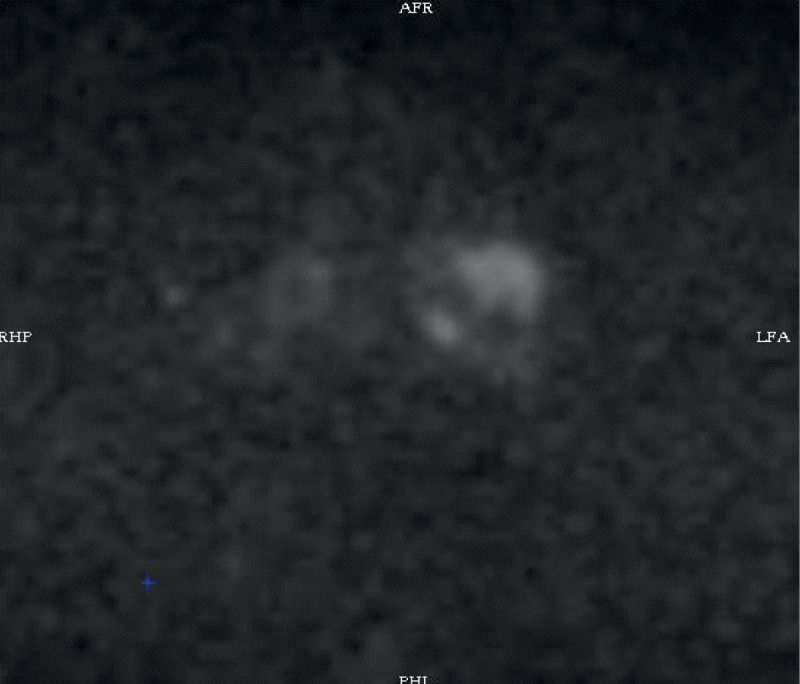


**Figure 2:**
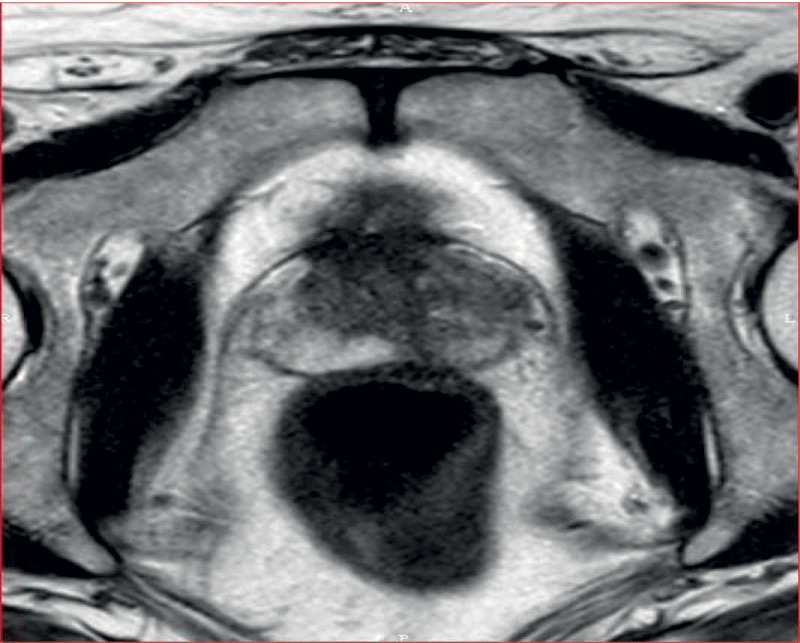


**Figure 3:**
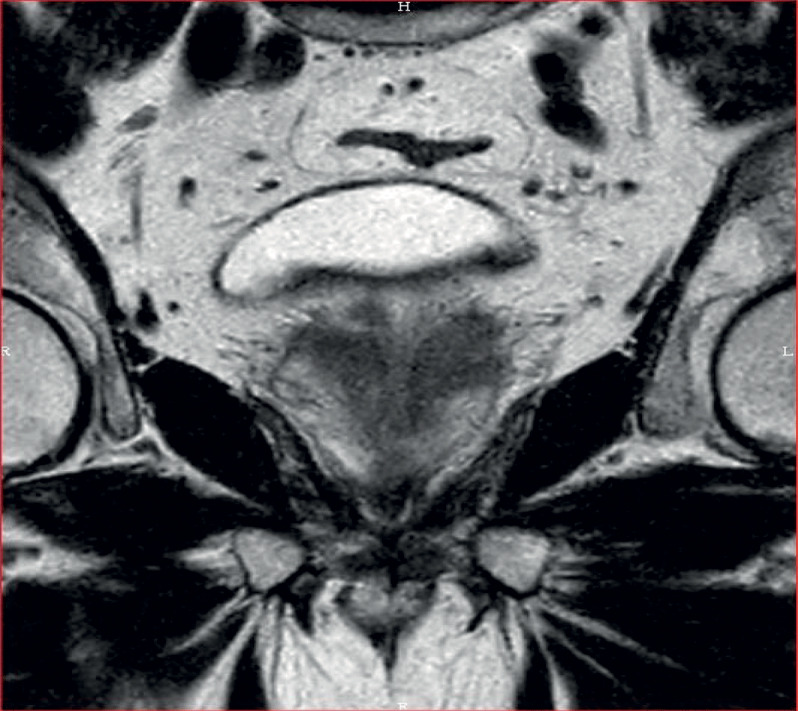


The patient is a 66-year-old man. He was treated 22 months ago for bladder urothelial neoplasm (pT1G3) by resection and topic bacilli Calmette-Guerin (BCG) instillations (Oncotice). At follow-up consultation, the digital rectal exam (DRE) revealed firmer prostatic left lobe and trans-rectal ultrasound showed a suspect hypoechogenic lesion at the left prostatic base. Prostatic-specific-antigen (PSA) level was slightly increased (4.77 ng/ml). A multi-parametric magnetic resonance imaging (mp-MRI) of the prostate revealed two focal lesions in the left peripheral zone. Both presented nodular high signal intensity on b1500 diffusion-weighted imaging (Figure 1), low apparent diffusion coefficient (ADC) value, T2 hypointensity, and pathological enhancement curves characterized by early, high level and with progressive wash-out (type III). The main lesion measured 15 mm and showed anterior capsular effraction (Figure 2), resulting in a score of 5 in the PIRADS v2.1 scale. The remaining lesion was smaller, medial, posterior and basal as compared to the first one, extending to the left seminal vesicle (Figure 3), which was collapsed. There was no associated lymph node enlargement. Guided biopsies were performed and led to the definitive diagnosis of granulomatous prostatitis (GP).

## Comment

Granulomatous prostatitis is a well-known but uncommon condition representing only 3.3% of all benign inflammatory lesions of the prostate. The incidence in the general population varies between 0.5 and 1.86% (1). Mean age of presentation is 61 years. GP may be idiopathic (nonspecific) or secondary to multiple causes including some specific urinary tract infective agents (Koch Bacillus - intravesical BCG therapy, treponema pallidum, some viruses and fungi), iatrogenic (surgery, biopsy, radiotherapy), malacoplakia associated with recurrent infection or other systemic granulomatous diseases like sarcoidosis. Idiopathic GP remains the most frequent type, seen in 69 to 77.7% of all cases [1].

At medical inspection, DRE can reveal a stiffer area. PSA may be elevated, and the patient can show hematuria. All these findings may also be present in prostate cancer (PCa). Moreover, some studies reported the coexistence of GP and carcinoma in 10–14% of cases [1].

On mp-MRI, GP appearances may completely overlap those of prostatic adenocarcinoma, showing nodular shape, low signal on T2-weighted imaging, high signal intensity on diffusion weighted imaging and low ADC. There is no defined ADC value which can differentiate both pathologies. Perfusion study may also be confounding. Nevertheless, some patients with GP present with a rim-enhancement related to abscess or caseous formation.

Clinical information is the key to evoking this diagnosis in case of secondary GP. Treatment should be adapted to the specific cause. In idiopathic GP, conservative and symptomatic treatment is administrated followed by surgery if ineffective.
